# Crystal structure of dihydroneopterin aldolase from *Mycobacterium tuberculosis* associated with 8-mercaptoguanine, and development of novel S8-functionalized analogues as inhibitors: Synthesis, enzyme inhibition, *in vitro* toxicity and antitubercular activity

**DOI:** 10.1080/14756366.2024.2388207

**Published:** 2024-08-14

**Authors:** Alexia de Matos Czeczot, Mauro Neves Muniz, Marcia Alberton Perelló, Éverton Edésio Dinis Silva, Luís Fernando Saraiva Macedo Timmers, Andresa Berger, Laura Calle Gonzalez, Guilherme Arraché Gonçalves, Sidnei Moura, Pablo Machado, Cristiano Valim Bizarro, Luiz Augusto Basso

**Affiliations:** a Centro de Pesquisas em Biologia Molecular e Funcional, Instituto Nacional de Ciência e Tecnologia em Tuberculose, Escola de Ciências da Saúde e da Vida, Pontifícia Universidade Católica do Rio Grande do Sul, Porto Alegre, Brazil; b Programa de Pós-Graduação em Medicina e Ciências da Saúde, Escola de Medicina, Pontifícia Universidade Católica do Rio Grande do Sul, Porto Alegre, Brazil; c Programa de Pós-Graduação em Biotecnologia, Universidade do Vale do Taquari, Lajeado, Brazil; d Programa de Pós-Graduação em Biologia Celular e Molecular, Escola de Ciências da Saúde e da Vida, Pontifícia Universidade Católica do Rio Grande do Sul, Porto Alegre, Brazil; e Laboratório de Biotecnologia de Produtos Naturais e Sintéticos, Instituto de Biotecnologia, Universidade de Caxias do Sul, Caxias do Sul, Rio Grande do Sul, Brazil

**Keywords:** Dihydroneopterin aldolase inhibitors, 8-mercaptoguanine, SAR, *Mycobacterium tuberculosis*

## Abstract

The crystallographic structure of the FolB enzyme from *Mycobacterium tuberculosis* (*Mt*FolB), complexed with its inhibitor 8-mercaptoguanine (8-MG), was elucidated at a resolution of 1.95 Å. A novel series of S8-functionalized 8-MG derivatives were synthesised and evaluated as *in vitro* inhibitors of dihydroneopterin aldolase (DHNA, EC 4.1.2.25) activity of *Mt*FolB. These compounds exhibited IC_50_ values in the submicromolar range. Evaluation of the activity for five compounds indicated their inhibition mode and inhibition constants. Molecular docking analyses were performed to determine the enzyme-inhibitor intermolecular interactions and ligand conformations upon complex formation. The inhibitory activities of all compounds against the *M. tuberculosis* H37Rv strain were evaluated. Compound **3e** exhibited a minimum inhibitory concentration in the micromolar range. Finally, Compound **3e** showed no apparent toxicity in both HepG2 and Vero cells. The findings presented herein will advance the quest for novel, specific inhibitors targeting *Mt*FolB, an attractive molecular target for TB drug development.

## Introduction

Tuberculosis (TB) is an infectious disease, which affects more than 10 million people every year. In 2022, the World Health Organisation (WHO) estimated 1.3 million of deaths from TB^1^. Numerous efforts have been made to combat this disease, and in 2014, the End TB strategy was adopted with the goal of reducing TB incidence by 80% and TB-related deaths by 90% by 2030, in comparison to 2015 levels^2^. Unfortunately, the COVID-19 pandemic had a negative impact on TB diagnosis and treatment in 2020 and 2021, reversing the progress made in previous years^3^. However, in 2022, there was a global recovery in the number of people diagnosed with TB and receiving treatment. Despite this progress, TB remains the major cause of deaths associated with antimicrobial resistance. Consequently, new approaches are required to control the spread of TB and mitigate its impact on the high number of patients and deaths^1^.

**Figure 1.**
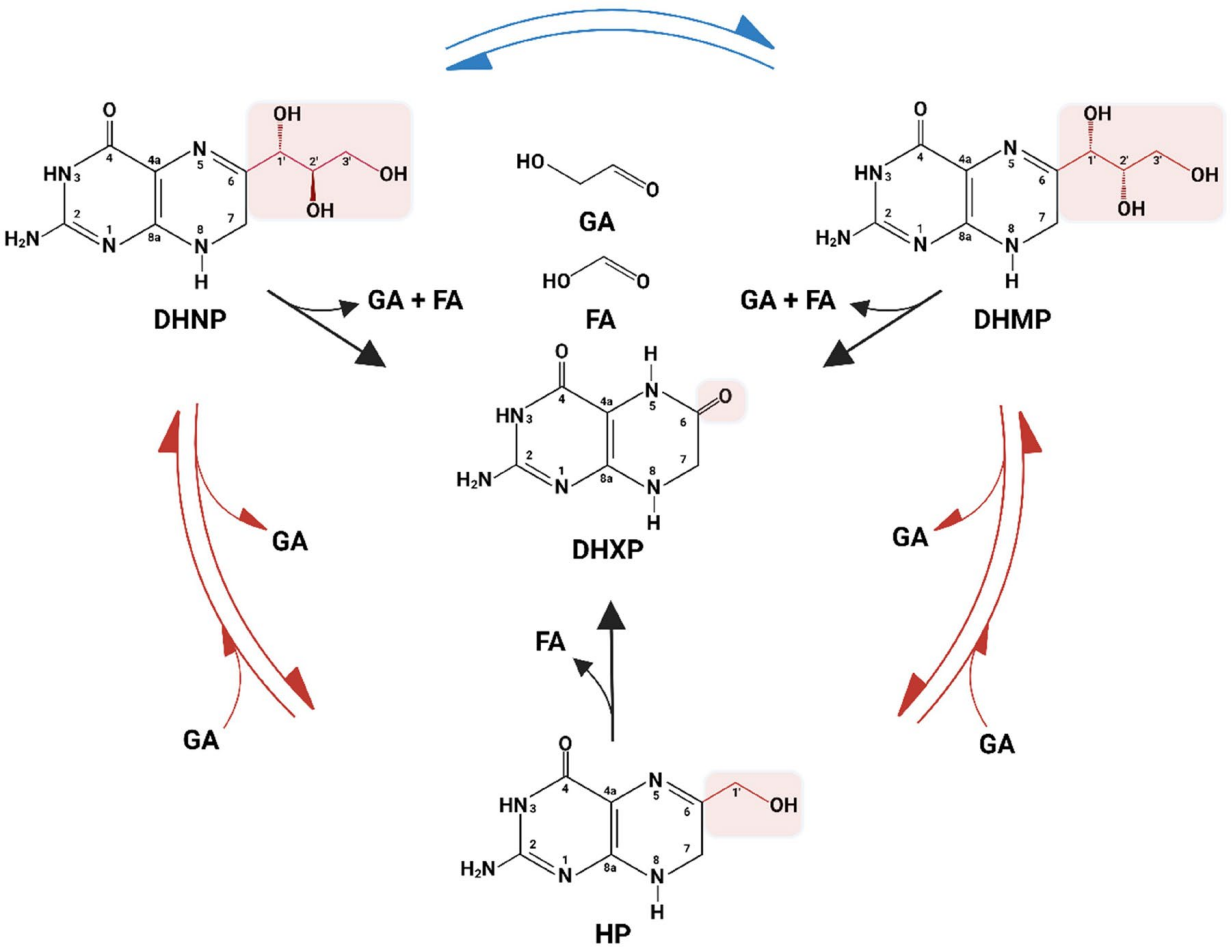
Aldolase, epimerase, and oxygenase reactions catalysed by *Mt*FolB. 7,8-dihydroneopterin (DHNP) and 7,8-dihydromonapterin (DHMP) are interconverted by the epimerase activity of *Mt*FolB (blue arrows). Both DHNP and DHMP are reversibly converted to 6-hydroxymethyl-7,8-dihydropterin (HP) and glycolaldehyde (GA) by *Mt*FolB aldolase activity (red arrows). Both DHNP and DHMP are also irreversibly converted to 7,8-dihydroxanthopterin (DHXP), GA and formic acid (FA), while HP are irreversibly converted to DHXP and FA by the *Mt*FolB oxygenase activity (black arrows).

The development of new anti-TB drugs that are effective against the growth of bacilli and act upon novel molecular targets represents a promising strategy^4^. The FolB enzyme from *Mycobacterium tuberculosis* (*Mt*FolB) is a potential target for anti-TB drugs due to its essential role in the survival of the bacillus and the absence of FolB homologues in humans^5^. *Mt*FolB exhibits three activities: aldolase, epimerase, and oxygenase. As an aldolase, this enzyme catalyses the reversible conversion of 7,8-dihydroneopterin (DHNP) to 6-hydroxymethyl-7,8-dihydropterin (HP) and glycolaldehyde (GA) in the folate biosynthesis pathway (Figure 1)^6^.

In this context, we have previously synthesised 8-mercaptoguanine (8-MG) derivatives containing benzyl, arylethanone or acetamide groups attached at the S-8 atom position^7^. 8-MG and its derivatives contain a substructure (H_2_N-C-NH-C = O) common to the substrates, products, and intermediate of the *Mt*FolB reactions. The S8-functionalized derivatives of 8-MG were synthesised to evaluate the activity of the compounds with different substituents and molecular volumes. We have also determined the inhibitory activities of 8-MG and these S8-substituted derivatives against *Mt*FolB. Although we identified the first *Mt*FolB inhibitors, with activities whitin the submicromolar range, none of them was found to affect the growth of *M. tuberculosis in vitro*^7^. Therefore, the identification of *Mt*FolB inhibitors possessing antimycobacterial activity remains a key objective.

**Scheme 1.**
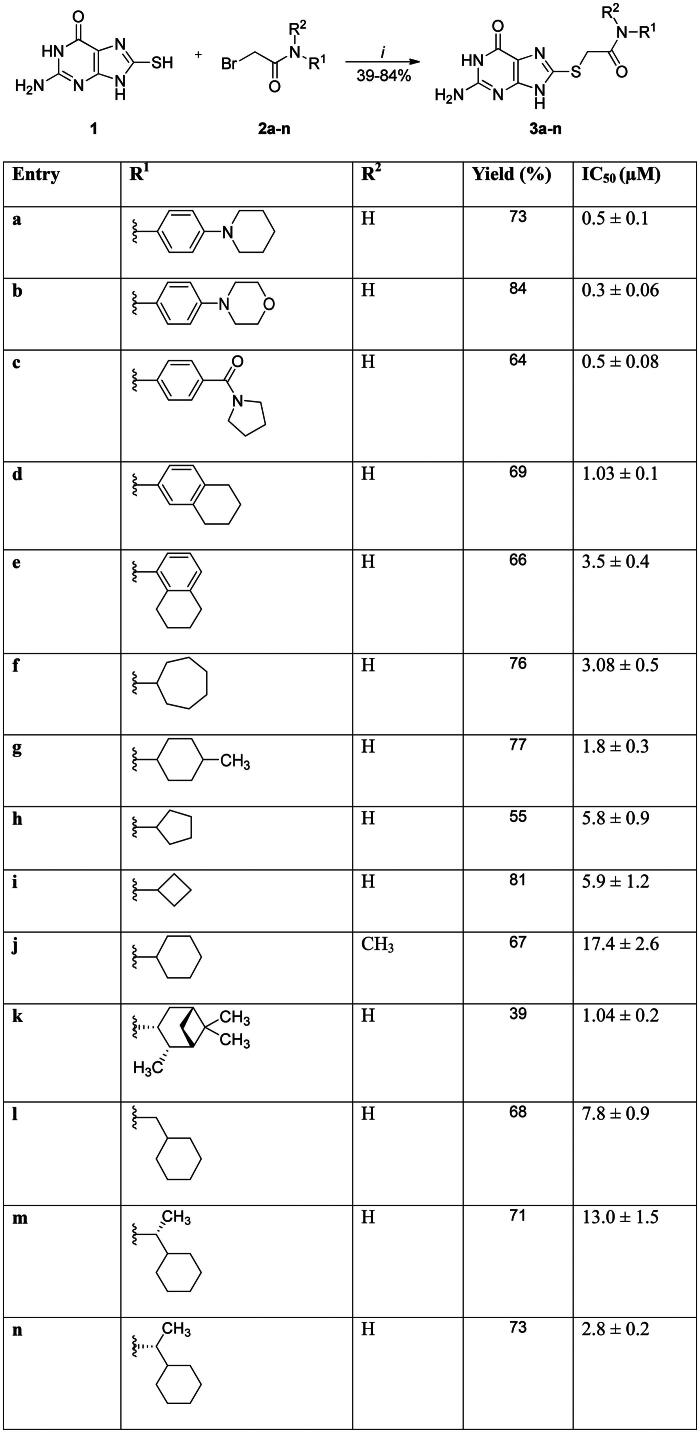
Reagents and reactants. Conditions: *i* = NaOH (0.5 M), EtOH, 25 °C, 2–6 h.

In this study, we determined the crystallographic structure of *Mt*FolB in complex with 8-MG. The quaternary structure of this complex was found to be in the octameric form, sharing the major structural changes previously observed for the octameric form of the *Mt*FolB bound to the product HP, that was proposed to stabilise the tetramer-tetramer interface and favour the oligomerisation of apo-tetramers^8^. Moreover, we synthesised 14 novel S8-functionalized analogues of 8-MG (Scheme 1) and assessed their inhibitory activity against *Mt*FolB enzyme by determining their IC_50_ values. We determined the mode and constant of inhibition for 5 compounds and conducted molecular docking to analyse their interactions with enzyme residues. Furthermore, we determined the minimum inhibitory concentration (MIC) for all compounds against the drug-susceptible *M. tuberculosis* H37Rv strain. Compound **3e** exhibited antimycobacterial activity, with a MIC value of 40 µg/mL (0.1 mM). Finally, we assessed the solubility of compound **3e** under three different conditions and evaluated its toxicity in cell viability experiments using both HepG2 and Vero cells.

## Materials and methods

### Expression and purification of MtFolB

The expression and purification of the recombinant enzyme *Mt*FolB was carried out as previously described^5^. Briefly, *Mt*FolB was purified using high performance liquid chromatography (HPLC) with the ÄKTA System (GE Healthcare^®^ Life Sciences, Pittsburgh, USA), using three chromatography columns: the anionic exchange column DEAE Sepharose CL6B (GE Healthcare^®^ Life Sciences, Pittsburgh, USA), the hydrophobic interaction column Butyl Sepharose High Performance HiLoad16/10 (GE Healthcare^®^ Life Sciences, Pittsburgh, USA) and the size exclusion column HiLoad Superdex 200 26/60 (GE Healthcare^®^ Life Sciences, Pittsburgh, USA).

### Crystallisation and data collection

Co-crystallisation of *Mt*FolB and the inhibitor 8-MG was performed in the space group *P*42_1_2. The ligand was dissolved in DMSO at concentration of 5 mg/mL. A solution containing *Mt*FolB and 8-MG was prepared by mixing 12 mg/mL of the enzyme with 10% of the ligand solution, obtaining a final concentration of 0.5 mM of 8-MG. Then, the solution was centrifuged at 16,000 g for 10 min at 4 °C, followed by incubation for approximately 2 h at 18 °C, before setting up the crystallisation conditions. Hanging drops were prepared mixing 1 µL of *Mt*FolB/8-MG solution and 1 µL of reservoir solution containing Bis-Tris 0.1 M pH 6.5 and NaCl 3 M. The crystals were cryoprotected with 10% of glycerol. X-ray diffraction data were collected at 1.542 Å wavelength using Synchrotron (Centro Nacional de Biologia Estrutural e Bioimagem (CENABIO), Universidade Federal do Rio de Janeiro (UFRJ, RJ, Brazil). The molecular replacement was conducted using Phaser Crystallographic Software^9^, with the crystallographic structure of *Mt*FolB complexed with 6-hydroxymethyl-7,8-dihydropterin (HP) (PDB ID 1NBU)^8^ as a template. The resulting data were processed using Phenix^10^ software. The interactions between *Mt*FolB and 8-MG were analysed using PoseView^11^. The PISA software^12^ was used to predict the probable quaternary structure of the *Mt*FolB/8-MG complex by assembling the macromolecular structure from X-ray crystallography data. An omit map of the bound 8-MG ligand was created by removing the ligand from the structure and undergoing 3 cycles of gradient energy minimisation using Phenix software.

### Synthesis

All reagents, chemicals and solvents were obtained from commercial sources and used as received, without any additional purification methods. Ethanol, dichlorometane and methanol were obtained from Química Moderna (Barueri, Brazil). Acetic acid (glacial), acetonitrile (high performance liquid chromatography (HPLC) grade), and methanol (HPLC grade) were obtained from Merck KGaA (Darmstadt, Germany). Sodium hydroxide was purchased from Êxodo científica (Sumaré, Brazil). Amines, bromoacetyl chloride, 4-(Dimethylamino)pyridine (DMAP) and DMSO-d6 were obtained from Sigma-Aldrich Corporation (Saint Louis, USA). Finally, 8-MG was obtained from Acros Organics B.V.B.A. (Geel, Belgium). The melting points of the synthesised compounds were accurately determined using a Microquímica MQAPF-302 melting point apparatus.^1^H and ^13^C Nuclear Magnetic Ressonance (NMR) spectra were obtained with an Avance III HD Bruker spectrometer (Bruker Corporation, Fällanden, Switzerland). This spectrometer operated at frequencies of 400 MHz for ^1^H NMR and 100 MHz for ^13^C NMR, utilising standard pulse sequences. The chemical shifts (δ) are reported in parts per million (ppm) and were referenced to dimethyl sulfoxide-d6 (DMSO-d6) as the solvent and tetramethylsilane (TMS) as the internal standard. High-resolution mass spectrometry (HRMS) analyses were conducted on Bruker Micro TOF-QII instrument, employing electrospray ionisation (ESI) at the University of Caxias do Sul (UCS). The relative purity of the molecules was assessed using a Dionex Ultimate 3000 High-Performance Liquid Chromatography (HPLC) system (Thermo Fischer Scientific Inc., Waltham, MA, USA). This system is equipped with a dual pump, an automatic injector, and a UV detector. Stock solutions of the compounds were prepared at a concentration of 1.0 mg/mL in a mixture of acetonitrile and methanol (1:1, v/v). These solutions were further diluted to a concentration of 0.5 mg/mL for the HPLC analysis.

#### General procedure for synthesis of compounds 3a–n

The synthesis of compounds 3a–n was carried out following previously established methods^7^^,^^13^. Briefly, 8-MG (1 mmol) was dissolved in 0.5 M NaOH (5 mL), forming a clear solution. To this, the corresponding bromoacetamides (1.2 mmol) was added in ethanol (1 mL). The reaction mixture was stirred for 2 to 6 h at a constant temperature of 25 °C. Subsequently, 5% acetic acid was added gradually until the pH reached 5. This adjustment in pH led to the formation of a precipitate, which was collected as an amorphous solid. When further purification was necessary, recrystallization was performed using methanol.

***2-((2-amino-6-oxo-6,9-dihydro-1H-purin-8-yl)thio)-N-(4-(piperidin-1-yl)phenyl)acetamide (3a)***. Yield: 73%; MP: 258–260 °C (dec); UHPLC: 97.2% (*t*_R_ = 12.18 min); ^1^H NMR (400 MHz, DMSO-*d*_6_) δ ppm 1.42 − 1.65 (m, 6H), 2.88 − 3.09 (m, 4H), 3.96 (s, 2H), 6.61 (s, 2H), 6.85 (d, J = 9.1 Hz, 2H), 7.43 (d, J = 9.1 Hz, 2H), 10.54 (s, 1H); ^13^C NMR (101 MHz, DMSO-*d*_6_) δ ppm 23.79, 25.22 (2 C), 36.24, 50.02 (2 C), 116.11 (2 C), 120.14 (2 C), 130.61, 143.84, 148.06, 153.27, 155.45, 155.89, 165.85, 173.67; FTMS (ESI) m/z: 400.1526 [M + H]^+^; calc. for C_18_H_22_N_7_O_2_S: 400.1550.

***2-((2-amino-6-oxo-6,9-dihydro-1H-purin-8-yl)thio)-N-(4-morpholinophenyl)acetamide (3b)***. Yield: 84%; MP: 326–328 °C (dec); UHPLC: 90.41% (*t*_R_ = 13.38 min); ^1^H NMR (400 MHz, DMSO-*d*_6_) δ ppm 3.03 (t, J = 4.5 Hz, 4H), 3.72 (t, J = 4.5 Hz, 4H), 4.05 (s, 2H), 6.35 (s, 2H), 6.89 (d, J = 8.5 Hz, 2H), 7.45 (d, J = 8.5 Hz, 2H), 10.28 (s, 1H); ^13^C NMR (101 MHz, DMSO-*d*_6_) δ ppm 36.17, 48.81 (2 C), 66.00 (2 C), 115.37 (2 C), 119.95 (2 C), 131,27, 144.44, 147.19, 152.92, 155.40, 156.29, 166.02, 173.69; FTMS (ESI) m/z: 402.1343 [M + H]^+^; calc. for C_17_H_20_N_7_O_3_S: 402.1343.

***2-((2-amino-6-oxo-6,9-dihydro-1H-purin-8-yl)thio)-N-(4-(pyrrolidine-1-carbonyl)phenyl)acetamide (3c)***. Yield: 64%; MP: 237–239 °C (dec); UHPLC: 96.59% (*t*_R_ = 14.15 min); ^1^H NMR (400 MHz, DMSO-*d*_6_) δ ppm 1.76 − 1.89 (m, 4H), 3.37 − 3.46 (m, 4H), 3.98 (s, 2H), 6.70 (s, 2H), 7.34 (s, 1H), 7.48 (d, J = 8.2 Hz, 2H), 7.67 (d, J = 8.0 Hz, 2H); ^13^C NMR (101 MHz, DMSO-*d*_6_) δ ppm 23.79, 25.93, 36.24, 45.90, 48.89, 118.02 (2 C), 128.01 (2 C), 131.50, 140.45, 151.13, 152.83, 155.85, 163.73, 167.54, 167. 76, 174.28; FTMS (ESI) m/z: 414.1335 [M + H]^+^; calc. for C_18_H_20_N_7_O_3_S: 414.1343.

***2-((2-amino-6-oxo-6,9-dihydro-1H-purin-8-yl)thio)-N-(5,6,7,8-tetrahydronaphthalen-2-yl)acetamide (3d)***. Yield: 69.2%; MP: 294–295 °C (dec); UHPLC: 94.43% (*t*_R_ = 15.74 min); ^1^H NMR (400 MHz, DMSO-*d*_6_) δ ppm 1.63 − 1.76 (m, 4H), 2.62 − 2.68 (m, 4H), 3.99 (s, 2H), 6.47 (s, 2H), 6.95 (d, J = 8.2 Hz, 1H), 7.26 (d, J = 8.3 Hz, 1H), 7.33 (s, 1H), 10.70 (s, 1H); ^13^C NMR (101 MHz, DMSO-*d*_6_) δ ppm 22.61, 22.74, 28.15, 28.89, 36.27, 115.57, 116.58, 119.19, 128.93, 131.57, 136.33, 136.70, 152.99, 155.29, 156.00, 166.24, 173.22; FTMS (ESI) m/z: 371.1231 [M + H]^+^; calc. for C_17_H_19_N_6_O_2_S: 371.1285.

***2-((2-amino-6-oxo-6,9-dihydro-1H-purin-8-yl)thio)-N-(5,6,7,8-tetrahydronaphthalen-1-yl)acetamide (3e)***. Yield: 66%; MP: 260–261 °C (dec); UHPLC: 91.41% (*t*_R_ = 15.01 min); ^1^H NMR (400 MHz, DMSO-*d*_6_) δ ppm 1.52 − 1.75 (m, 4H), 2.45 − 2.56 (m, 2H), 2.62 − 2.74 (m, 2H), 4.01 (s, 2H), 6.65 (s, 2H), 6.85 (d, J = 7.6 Hz, 1H), 7.03 (t, J = 7.8 Hz, 1H), 7.38 (d, J = 8.0 Hz, 1H), 9.92 (s, 1H).^13^C NMR (101 MHz, DMSO-*d*_6_) δ ppm 22.16, 22.33, 24.10, 29.18, 35.64, 115.79, 121.02, 124.89, 125.57, 129.74, 135.85, 137.25, 153.06, 155.50, 156.38, 167.08, 173.82; FTMS (ESI) m/z: 371.1239 [M + H]^+^; calc. for C_17_H_19_N_6_O_2_S: 371.1285.

***2-((2-amino-6-oxo-6,9-dihydro-1H-purin-8-yl)thio)-N-cycloheptylacetamide (3f)***. Yield: 76%; MP: 292–294 °C (dec); UHPLC: 90.12% (*t*_R_ = 14.91 min); ^1^H NMR (400 MHz, DMSO-*d*_6_) δ ppm 1.29 − 1.46 (m, 5H), 1.46 − 1.63 (m, 4H), 1.67 − 1.80 (m, 3H), 3.73 (tt, J = 8.2, 4.0 Hz, 1H), 3.77 (s, 2H), 6.19 (s, 2H), 8.02 (d, J = 7.8 Hz, 1H); ^13^C NMR (101 MHz, DMSO-*d*_6_) δ ppm 23.35 (2 C), 27.35 (2 C), 33.73 (2 C), 35.31, 49.73, 114.99, 143.11, 152.86, 154.69, 155.28, 166.08; FTMS (ESI) m/z: 337.1448 [M + H]^+^; calc. for C_14_H_21_N_6_O_2_S: 337.1441.

***2-((2-amino-6-oxo-6,9-dihydro-1H-purin-8-yl)thio)-N-(4-methylcyclohexyl)acetamide (3 g)***. Yield: 77%; MP: 282–284 °C (dec); UHPLC: 91.07% (*t*_R_ = 15.37 min); ^1^H NMR (400 MHz, DMSO-*d*_6_) δ ppm 0.81 (dd, J = 31.1, 6.2 Hz, 3H), 0.87 − 1.00 (m, 1H), 1.11 (t, J = 12.8 Hz, 2H), 1.29 − 1.46 (m, 2H), 1.49 − 1.57 (m, 1H), 1.68 (dd, J = 41.8, 13.2 Hz, 3H), 3.35 − 3.48 (m, 1H), 3.79 (s, 2H), 6.81 (d, J = 18.4 Hz, 2H), 8.33 (dd, J = 19.0, 7.7 Hz, 1H); ^13^C NMR (101 MHz, DMSO-*d*_6_) δ ppm 23.69, 28.92, 29.06, 31.41, 32.09, 33.47, 35.49, 48.18, 153.33, 153.47, 155.74, 166.82, 167.46, 174.41; FTMS (ESI) m/z: 337.1397 [M + H]^+^; calc. for C_14_H_21_N_6_O_2_S: 337.1441.

***2-((2-amino-6-oxo-6,9-dihydro-1H-purin-8-yl)thio)-N-cyclopentylacetamide (3h)***. Yield: 55%; MP: 280–282 °C (dec); UHPLC: 91.56% (*t*_R_ = 13.34 min); ^1^H NMR (400 MHz, DMSO-*d*_6_) δ ppm 1.31 − 1.43 (m, 2H), 1.43 − 1.54 (m, 3H), 1.56 − 1.65 (m, 2H), 1.69 − 1.81 (m, 1H), 3.73 (s, 2H), 3.91 − 4.06 (m, 1H), 6.53 (s, 2H), 8.42 − 8.48 (m, 1H); ^13^C NMR (101 MHz, DMSO-*d*_6_) δ ppm 23.02 (2 C), 31.89 (2 C), 35.16, 50.46, 115.75, 144.59, 152.81, 155.54, 167.35, 173.99; FTMS (ESI) m/z: 309.1124 [M + H]^+^; calc. for C_12_H_17_N_6_O_2_S: 309.1128.

***2-((2-amino-6-oxo-6,9-dihydro-1H-purin-8-yl)thio)-N-cyclobutylacetamide (3i)***. Yield: 81%; MP: 285–287 °C (dec); UHPLC: 92.62% (*t*_R_ = 13.58 min); ^1^H NMR (400 MHz, DMSO-*d*_6_) δ ppm 1.51 − 1.67 (m, 2H), 1.79 − 1.96 (m, 2H), 2.04 − 2.20 (m, 2H), 3.77 (s, 2H), 4.00 − 4.25 (m, 1H), 6.91 (s, 2H), 8.86 (d, J = 7.7 Hz, 1H); ^13^C NMR (101 MHz, DMSO-*d*_6_) δ ppm 14.73, 30.13 (2 C), 35.29, 44.23, 115.87, 153.30, 156.05, 156.73, 166.87, 174.98; FTMS (ESI) m/z: 295.0978 [M + H]^+^; calc. for C_11_H_15_N_6_O_2_S: 295.0972.

***2-((2-amino-6-oxo-6,9-dihydro-1H-purin-8-yl)thio)-N-cyclohexyl-N-methylacetamide (3j)***. Yield: 67%; MP: 272–274 °C (dec); UHPLC: 91.13% (*t*_R_ = 14.43 min); ^1^H NMR (400 MHz, DMSO-*d*_6_) δ ppm 0.97 − 1.16 (m, 1H), 1.16 − 1.35 (m, 1H), 1.34 − 1.54 (m, 3H), 1.53 − 1.82 (m, 5H), 2.80 (d, J = 71.4 Hz, 3H), 3.55 − 3.72 (m, 1H), 4.24 (d, J = 18.6 Hz, 2H), 6.91 (s, 2H); ^13^C NMR (101 MHz, DMSO-*d*_6_) δ ppm 24.01, 25.05, 25.31, 27.13, 29.11, 30.17, 36.26, 52.47, 56.16, 115.29, 153.61, 155.75, 165.46, 166.68, 174.83; FTMS (ESI) m/z: 337.1436 [M + H]^+^; calc. for C_14_H_21_N_6_O_2_S: 337.1441.

***2-((2-amino-6-oxo-6,9-dihydro-1H-purin-8-yl)thio)-N-((1S,2S,3R,5R)-2,6,6-trimethylbicyclo [3.1.1] heptan-3-yl)acetamide (3k)***. Yield: 39%; MP: 289–291 °C (dec); UHPLC: 93.07% (*t*_R_ = 15.73 min); ^1^H NMR (400 MHz, DMSO-*d*_6_) δ ppm 0.84 − 1.07 (m, 6H), 1.18 (s, 3H), 1.48 (ddt, J = 13.4, 7.0, 3.6 Hz, 1H), 1.74 (s, 2H), 1.85 (qd, J = 7.1, 2.5 Hz, 2H), 2.24 − 2.36 (m, 2H), 3.81 (s, 2H), 3.96 − 4.08 (m, 1H), 6.83 (s, 2H), 8.58 (d, J = 8.4 Hz, 1H); ^13^C NMR (101 MHz, DMSO-*d*_6_) δ ppm 20.62, 23.15, 27.85, 33.99, 35.43, 36.00, 38.08, 41.00, 44.27, 47.19, 47.22, 115.70, 153.43, 155.85, 156.20, 167.21, 174.76; FTMS (ESI) m/z: 377.1745 [M + H]^+^; calc. for C_17_H_25_N_6_O_2_S: 377.1754.

***2-((2-amino-6-oxo-6,9-dihydro-1H-purin-8-yl)thio)-N-(cyclohexylmethyl)acetamide (3 l)***. Yield: 68%; MP: 262–263 °C (dec); UHPLC: 90.14% (*t*_R_ = 15.08 min); ^1^H NMR (400 MHz, DMSO-*d*_6_) δ ppm 0.81 (q, J = 13.5, 11.7 Hz, 2H), 0.95 − 1.20 (m, 2H), 1.32 (dtd, J = 15.6, 7.6, 4.1 Hz, 1H), 1.49 − 1.72 (m, 6H), 2.91 (t, J = 6.5 Hz, 2H), 3.81 (s, 2H), 6.79 (s, 2H), 8.37 (t, J = 5.8 Hz, 1H); ^13^C NMR (101 MHz, DMSO-*d*_6_) δ ppm 25.38 (2 C), 25.95, 30.23 (2 C), 35.23, 37.35, 45.13, 115.65, 153.37, 155.76, 156.19, 167.78, 174.53; FTMS (ESI) m/z: 337.1404 [M + H]^+^; calc. for C_14_H_21_N_6_O_2_S: 337.1441.

***(S)-2-((2-amino-6-oxo-6,9-dihydro-1H-purin-8-yl)thio)-N-(1-cyclohexylethyl)acetamide (3 m)***. Yield: 71%; MP: 294–296 °C (dec); UHPLC: 91.66% (*t*_R_ = 14.70 min); ^1^H NMR (400 MHz, DMSO-*d*_6_) δ ppm 0.84 (dtd, J = 12.4, 9.2, 3.5 Hz, 2H), 0.97 (d, J = 6.8 Hz, 3H), 0.97 − 1.17 (m, 3H), 1.21 (tdq, J = 13.2, 6.7, 3.2 Hz, 1H), 1.59 (q, J = 15.1, 13.5 Hz, 5H), 3.58 (dq, J = 13.4, 6.9 Hz, 1H), 3.81 (q, J = 15.4, 13.6 Hz, 2H), 6.35 (s, 2H), 7.96 (d, J = 8.6 Hz, 1H); ^13^C NMR (101 MHz, DMSO-*d*_6_) δ ppm 17.50, 25.70 (2 C), 25.91, 28.38, 28.65, 35.47, 42.34, 48.98, 117.10, 140.64, 153.30, 153.44, 155.61, 166.56; FTMS (ESI) m/z: 351.1596 [M + H]^+^; calc. for C_15_H_35_N_6_O_2_S: 351.159

***(R)-2-((2-amino-6-oxo-6,9-dihydro-1H-purin-8-yl)thio)-N-(1-cyclohexylethyl)acetamide (3n)***. Yield: 73%; MP: 289–291 °C (dec); UHPLC: 96.76% (*t*_R_ = 14.73 min); ^1^H NMR (400 MHz, DMSO-*d*_6_) δ ppm 0.87 (tdd, J = 14.1, 10.2, 2.8 Hz, 2H), 0.97 (d, J = 6.7 Hz, 3H), 1.00 − 1.17 (m, 3H), 1.25 (s, 1H), 1.48 − 1.71 (m, 5H), 3.48 − 3.67 (m, 1H), 3.77 (q, 2H), 6.44 (s, 2H), 8.03 (d, J = 8.6 Hz, 1H); ^13^C NMR (101 MHz, DMSO-*d*_6_) δ ppm 17.13, 25.40 (2 C), 25.63, 28.11, 28.40, 35.13, 42.11, 48.75, 115.50, 143.85, 152.87, 155.19, 156.01, 166.94, 173.54; FTMS (ESI) m/z: 351.1589 [M + H]^+^; calc. for C_15_H_23_N_6_O_2_S: 351.1598.

### In vitro MtFolB inhibition assays

The 14 S8-substituted 8-MG compounds synthesised were evaluated as inhibitors of *Mt*FolB using the continuous fluorescence-based enzyme activity method previously described^7^. These assays were performed on RF-5301 spectrofluorophotometer (Shimadzu) with an excitation wavelength of 365 nm and fluorescence emission at 525 nm, monitoring for 6 min the conversion of 7,8-dihydroneopterin (DHNP) to 6-hydroxymethyl-7,8-dihydropterin (HP) and glycolaldehyde (GA) by means of an increase in fluorescence due to HP formation. The standard reaction was carried out at 25 mM Tris, 50 mM NaCl, 5% glycerol pH 8.0 at 25 °C. For each reaction condition, we performed control reactions including (1) a buffer-only control; (2) a buffer + DHNP (substrate) + DMSO/inhibitor control (no enzyme), and (3) a buffer + enzyme control (neither DHNP nor DMSO/inhibitor). The fluorescence values from control (1) were added, and those from controls (2) and (3) were subtracted from the reaction fluorescence to provide just the fluorescence of the reaction product^7^. The concentration of the inhibitor necessary to reduce 50% of the enzyme activity (IC_50_)^14^ was determined for each synthesised compound to provide its potency of inhibition. The IC_50_ values were obtained by determining the initial velocity curves of enzymatic reactions using 0.3 µM of *Mt*FolB, 1.5 µM of DHNP and different concentrations of the synthesised compounds (dissolved in DMSO) until enzymatic activity reaches zero. The maximal concentration of DMSO in this assay was 10% and the concentration ranges of each compound were: **3a** 0.05 − 1.5 µM; **3b** and **3c** 0.05 − 2.5 µM; **3d** 0.05 − 3 µM; **3e** and **3 l** 1 – 20 µM; **3f** 1.5 – 10 µM; **3 g** 0.5 – 5 µM; **3h** 1.5 – 12 µM; **3i** 1 – 15 µM; **3j** and **3 m** 2 – 40 µM; **3k** 0.2 – 5 µM; **3n** 0.5 − 7.5 µM. The maximal rate of enzymatic reaction at each condition was determined in control reactions performed in the absence of inhibitor and presence of 10% DMSO at the same conditions. The IC_50_ values were estimated using Equation (1), where *V*_i_ and *V*_0_ are, respectively, the reaction velocity in the presence and in the absence of inhibitor (I):

(1) $$\frac{V_{i}}{V_{0}}=\frac{1}{1+\frac{\left[I\right]}{{\text{IC}}_{50}}}$$

The time-dependent inhibitory activity was evaluated preincubating 0.3 µM of the *Mt*FolB (final concentration) with a fixed inhibitor concentration defined based on IC_50_ value for each compound (Scheme 1) (final concentration of 0.5 µM for **3a**; 0.35 µM for **3b**; 0.55 µM for **3c**; 1 µM for **3d** and **3k**; 3 µM for **3e**, **3f** and **3n**; 2 µM for **3 g**; 6 µM for **3h** and **3i**; 15 µM for **3j**; 8 µM for **3 l**; and 12 µM for **3 m**), then aliquots were taken after 5, 10, 20 and 40 min of preincubation and added to the reaction mixture described above. The percentage of inhibition was calculated after monitoring change in the initial velocity with time. As a control, *Mt*FolB was preincubated with DMSO at a final concentration of 10% and added to the reaction mixture.

The mode of inhibition and the inhibition constants (*K*_i_ and/or *K*_is_) were defined for the compounds **3a**-**e**. There are three categories for the inhibition mode: competitive, where inhibitor and substrate binding are mutually exclusive; non-competitive, if the inhibitor can bind to both the free enzyme and the enzyme-substrate complex; or uncompetitive, when the inhibitor binds only to the enzyme-substrate complex^14^. For each compound, 3 curves were performed with fixed-varied inhibitor concentration and varying the DHNP concentration until saturation of enzyme activity. For the compound **3a** the fixed-varied concentrations were 0.2 μM, 0.5 μM and 0.8 μM (varying DHNP 0.5 – 10 μM). For the compound **3b** the fixed-varied concentrations were 0.1 µM (varying DHNP 0.2 – 10 µM); 0.3 μM (varying DHNP 0.5 – 10 μM) and 0.6 μM (varying DHNP 0.5 – 15 μM). For the compound **3c** the fixed-varied concentrations were 0.3 μM, 0.6 μM and 0.9 μM (varying DHNP 0.5 – 10 μM). For the compound **3d** the fixed-varied concentrations were 0.5 μM, 1 μM (varying DHNP 0.5 – 10 μM) and 2.5 μM (varying DHNP 1 – 10 μM). For the compound **3e** the fixed-varied concentrations were 1.4 µM, 3.5 µM (varying DHNP 0.5 – 10 µM) and 7 µM (varying DHNP 1 – 10 µM). Furthermore, we performed a control experiment with 10% DMSO in the absence of inhibitors varying the DHNP concentration until saturation (0.2 – 10 µM). The straight-line patterns obtained were used to define the mode of inhibition. The diagnostic signature of a competitive inhibitor is a set of lines that intersect on the *y*-axis, for non-competitive inhibitors the pattern of intersection is to the left of the *y*-axis and for uncompetitive inhibitors the lines are parallel to each other^14^. The inhibition constants were calculated using Equation (2) for competitive inhibitors or Equation (3) for non-competitive inhibitors, where [I] is the inhibitor concentration, [S] is the substrate concentration, *K*_M_ is the Michaelis-Menten constant, V_max_ is the maximum velocity, *K*_is_ is the inhibition constant for the enzyme-substrate complex, and *K*_ii_ is the inhibition constant for the enzyme-substrate-inhibitor complex.

(2) $$\frac{1}{v_{0}}=\frac{K_{M}}{V_{\text{max}}}\left(1+\frac{\left[I\right]}{K_{\text{is}}}\right)\frac{1}{\left[S\right]}+\frac{1}{V_{\text{max}}}$$

(3) $$\frac{1}{v_{0}}=\frac{K_{M}}{V_{\text{max}}}\left(1+\frac{\left[I\right]}{K_{\text{is}}}\right)\frac{1}{\left[S\right]}+\frac{1}{V_{\text{max}}}\left(1+\frac{\left[I\right]}{K_{\text{ii}}}\right)$$

### Molecular docking studies

The prediction of the most favourable poses of the synthesised compounds into the active site of *Mt*FolB and the estimated binding energy were obtained by molecular docking. The crystallographic structure of *Mt*FolB complexed with 8-mercaptoguanine (8-MG) obtained in this study (section Crystallisation and data collection) was used in these efforts. The *Mt*FolB structure was aligned to the PDB structure 1NBU using PyMOL v 2.5 software^15^ and prepared adding all hydrogens atoms and removing the ligand from chain E using Gold v2023.2^16^. Before conducting the docking experiments, a redocking procedure was performed to establish the docking protocol that accurately reproduces the *in vitro* experimental data in these simulations, with the value of root-mean-square deviation (RMSD) < 1.0 Å. The redocking was carried out using Gold v2023.2 with GOLD default parameters. The extracted heteroatoms 8-MG from chain E of the crystallographic structure of *Mt*FolB-8-MG complex corresponds to the ligand and the reference ligand, and the binding site was defined within 10 Å radius of the coordinates × 21.339 *y* − 24.725 *z* 9.105, which corresponds to the nitrogen atom 69 from 8-MG. The GoldScore and ChemPLP were the score and rescore function, respectively, the GA runs was 20. The docking experiments were performed with the same protocol of the redocking using the prepared *Mt*FolB crystallographic structure. The ligand structures were drawn on Chemdraw (RRID:SCR_016768), then 3D structures were generated and prepared using Avogadro v1.1.1.^17^ The hydrogens atoms for pH 8 were added in the molecules, because it is the condition used during enzyme inhibition studies (section *In vitro Mt*FolB inhibition assays), and the MMFF94 force field^18^ was then applied. The protein complexes were visualised in PyMOL v2.5^15^ and the interactions were analysed using PoseView^11^.

### Determination of minimum inhibitory concentration

The synthesised compounds were evaluated for their ability to inhibit the growth of *M. tuberculosis* H37Rv strain (ATCC 27294) using the colorimetric resazurin reduction microplate assay (REMA) to determine the minimum inhibitory concentration (MIC)^19^. The MIC represents the lowest concentration of the compound that completely prevents visible growth of the bacillus^20^. Isoniazid (INH) and rifampicin (RIF) were the drug controls in this assay. The stock solutions for all compounds were prepared at 2 mg/mL in DMSO (Sigma-Aldrich) and stored at −20 °C. Working solutions were freshly prepared at the time of experiment, diluting the compounds in Difco^TM^ Middlebrook 7H9 broth (Becton, Dickinson and Company – BD) supplemented with 10% BBL^TM^ Middlebrook ADC enrichment (albumin, dextrose and catalase – BD) with DMSO at a final concentration of 5% at the solubility limit of each compound, without forming crystals. For each compound, we performed 10-point (for INH, RIF, **3d**, **3h**, **3k** and **3n**) or 6-point (for **3a-c**, **3e-g**, **3i**, **3j**, **3 l** and **3 m**) 2-fold serial dilutions in 100 µL of Middlebrook 7H9 medium with 10% Middlebrook ADC enrichment directly in 96-well plates. The concentrations ranged from 10 to 0.02 µg/mL for INH and **3d**; from 1.25 to 0.002 µg/mL for RIF; from 10 to 0.31 µg/mL for **3a**; from 40 to 1.25 µg/mL for **3b**, **3c**, **3e-g**, **3i**, **3j**, **3 l** and **3 m**; and from 40 to 0.08 µg/mL for **3h**, **3k** and **3n**. *M. tuberculosis* was cultured in Middlebrook 7H9 broth containing 10% BD Difco ^™^ BBL ^™^ Middlebrook OADC enrichment (oleic acid, albumin, dextrose, catalase), 0.05% Tween 80 (Sigma-Aldrich) and 0.2% glycerol (Sigma-Aldrich) at 37 °C to an optical density at 600 nm (OD_600_) of 0.6 to 0.8. Then, mycobacterial cultures were suspended by vortexing with sterile glass beads (1 mm) for 3 min and left to rest for 15 min. The supernatant was measured at OD_600_ and aliquots were stored at −80 °C. Bacterial cultures were then diluted to a theoretical OD_600_ of 0.006 in Middlebrook 7H9 broth supplemented with 10% Middlebrook ADC enrichment and transferred to 96-well plates (100 µL/well). The final concentration of DMSO in the assay was 2.5%. Growth controls without drugs and sterility controls without mycobacteria were included on each plate. The plates were covered, sealed with parafilm, and incubated at 37 °C for 7 days. A 60 µL solution of 0.01% resazurin (Sigma-Aldrich) was added on each well of the plate before a final incubation step at 37 °C for 48 h. The MIC values were obtained by determining the lower compound concentration at which no colour change from blue (growth inhibition) to pink (indicating growth) is observed. Three independent experiments were performed, and MIC values were considered the most frequent compound concentration that inhibited the visible growth of bacilli.

### Cell viability evaluation

Cell viability assays in the presence of compound **3e** were performed in both Vero (BCRJ code 0245) and HepG2 (BCRJ code 0291) cells using MTT (3-[4,5-dimethylthiazol-2-yl]-2,5 diphenyl tetrazolium bromide)^21^ and neutral red uptake (NRU)^22^ methods. Both cell lines were grown in Dulbecco’s Modified Eagle Medium (DMEM-Gibco, Grand Island, NY, USA) supplemented with 10% or 20% inactivated foetal bovine serum (FBS- Invitrogen) for Vero and HepG2, respectively, 1% penicillin (Gibco) and streptomycin (Gibco), and 0.1% fungizone (Gibco). The experiments were performed in 96-well plates. First, cells were seeded at 6 x 10^3^ (Vero) or 7 x 10^3^/well (HepG2) and incubated overnight at 37 °C under a 5% CO_2_ atmosphere. Then, the medium was removed and replaced with fresh medium containing the compound at a final concentration of 75 µM, 87.5 µM, or 100 µM with DMSO at a final concentration of 0.8%. Cells were incubated during 72 h at 37 °C under a 5% CO_2_ atmosphere. For the MTT assay, the medium was removed after the incubation and replaced with MTT reagent (Sigma-Aldrich) at 5 mg/mL, and then the cells were incubated for 4 h. DMSO was used to solubilise formazan crystals. The absorbance was measured at 570 nm using microplate reader (EZ Read 400 microplate reader, Biochrom, Cambridge, UK). Precipitated purple formazan crystals were directly proportional to the number of live cells with mitochondrial metabolism. For the NRU assay, after the incubation with the compound, the media was removed and 200 µL of neutral red dye solution (Sigma-Aldrich) at 25 µg/mL prepared in serum-free medium was added to each well. The plate was then incubated for 3 h at 37 °C under 5% CO_2_. After this time, cells were washed twice with PBS, followed by incubation with 100 μL of a desorb solution (ethanol/acetic acid/water, 1:50:49) for 30 min, with gentle shaking to extract neutral red dye from the viable cells. The absorbance was measured at 562 nm using microplate reader (EZ Read 400 microplate reader, Biochrom, Cambridge, UK). Treated control wells (0.8% DMSO) were used as references for maximum cell viability (100%). Three independent experiments were performed, and the data were expressed as the means of cell viability ± the standard error of the means. Statistical analysis was performed using one-way ANOVA analysis of variance followed by Dunett’s Multiple Comparison Test post-test using GraphPad Prism 9.0.0 (San Diego, CA, USA).

### Solubility assay

The solubility of the compound **3e** was evaluated under three pH conditions: pH 1.2 (HCl 0.1 M); pH 7.4 (PBS 1X); and pH 9.1 (NH_4_HCO_3_ 0.1 M). For this assay, we prepared a suspension of compound **3e** at 1 mg/mL in each buffer solution by vortexing it for 1 min. Then, the suspension was kept under constant shaking for 4 h at 25 °C. Subsequently, the suspension was centrifuged at 13,000 rpm for 15 min at 25 °C. The supernatant was quantified by ultra-high-performance liquid chromatography (UHPLC) using single-point calibration of a known concentration of the compound in DMSO. The experiment was performed in triplicate.

## Results and discussion

**Figure 2.**
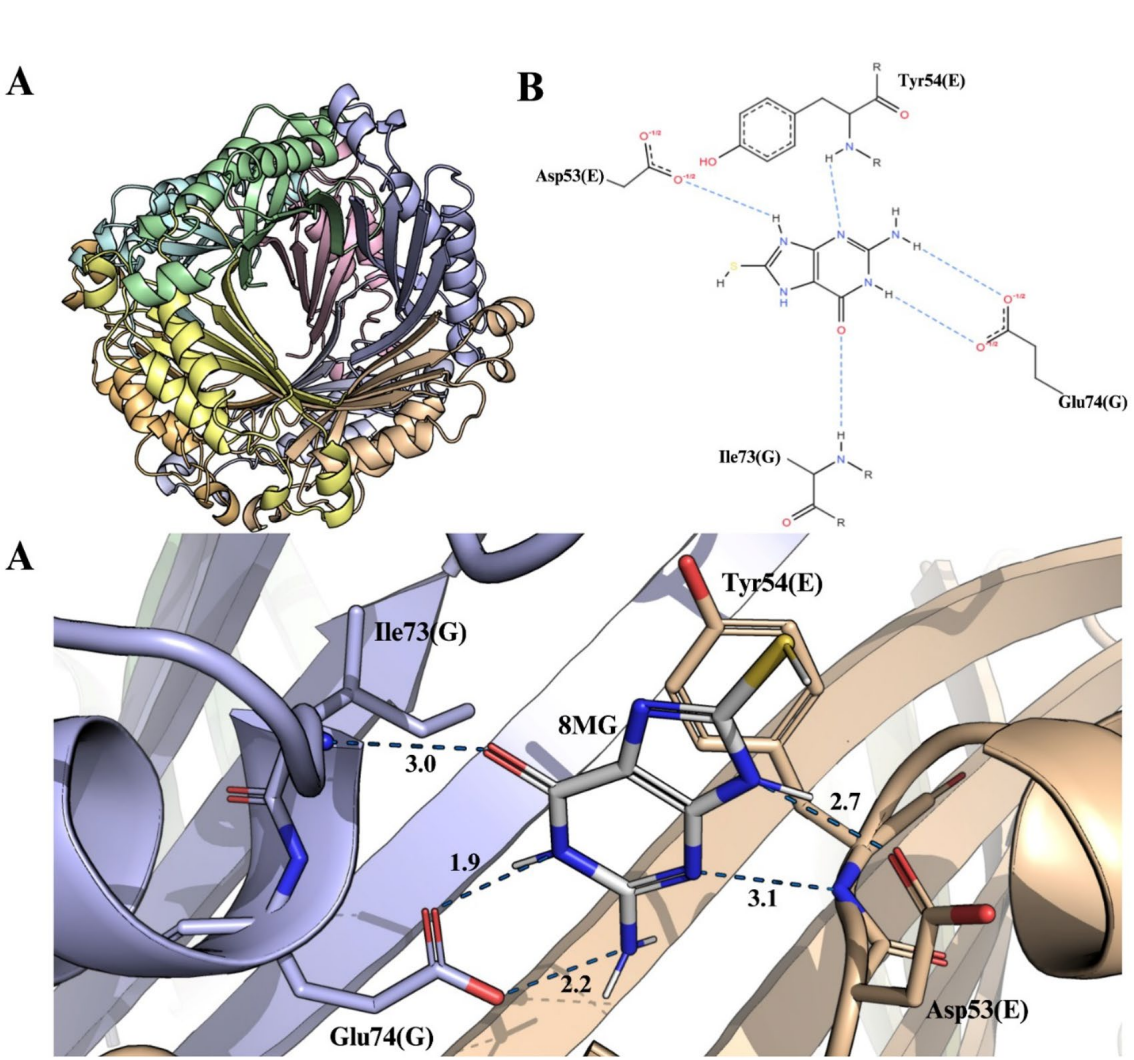
Three-dimensional structure of octameric *Mt*FolB complexed with 8-MG and their 2D and 3D interactions representation. (A) The 3D crystallographic structure of *Mt*FolB complexed with 8-MG revels that *Mt*FolB forms an octamer upon interaction with 8-MG. *Mt*FolB is represented as cartoon, with each chain is represented in a distinct colour: A (green), B (cyan), C (magenta), D (yellow), E (salmon), F (gray), G (slate blue), and H (orange). 8-MG is present between each monomer and is represented in stick model. (B) 2D interaction diagrams between 8-MG and *Mt*FolB residues, with hydrogen bonds (black) and π–π stacking interactions (green) shown in dashed lines. (C) An enlarged view of 3D interaction with 8-MG. The main residues that interact by polar contacts with 8-MG are shown as sticks. 3D figures were generated using PyMOL v2.5 and 2D figures using PoseView^11^.

The crystallographic structure of *Mt*FolB complexed with 8-MG was solved (Figure 2). The crystals diffracted at 1.95 Å resolution and were resolved in the space group *P*42_1_2, with two *Mt*FolB chains in each asymmetric unit. The data collection and structure refinement are summarised in Table 1. To verify the presence of the ligand 8-MG in the resolved structure, the omit map of this ligand was generated. Fo-Fc electron density (green) is contoured to 2 σ, indicating evidence of a bound ligand (Figure S1).

**Table 1.** X-ray diffraction data collection and atomic refinement for *Mt*FolB complexed with 8-mercaptoguanine crystallographic structure.

X-ray wavelenght (Å)	1.542
Space group	P42_1_2
Unit-cell parameters (a, b, c (Å)/α = β = ɣ (°))	81.386, 81.386, 74.862/ 90
Resolution range (Å)	19.33 − 1.95 (2.02 − 1.95)
Total reflections	34614 (3210)
Unique reflections	18885 (1847)
Multiplicity	1.8 (1.7)
Completeness (%)	97.95 (99.73)
*I* /σ	11.45 (2.96)
R_merge_	0.05041 (0.217)
R_meas_	0.07129 (0.3069)
R_pim_	0.05041 (0.217)
CC1/2	0.997 (0.939)
CC*	0.999 (0.984)
R_work_	0.2048 (0.2655)
R_free_	0.2131 (0.2766)
CC(work)	0.953 (0.909)
CC(free)	0.962 (0.873)
Solvent	282
Residues	236
Observed RMSD from the ideal geometry	
Bond lenghts (Å)	0.009
Bond angles (°)	1.09
Ramachandran Plot	
Residues in most favoured regions (%)	99.14
Residues in additionally allowed regions (%)	0.86
Ramachandran outliers (%)	0.00
Rotamers outliers (%)	0.00

We found an octamer as the most likely quaternary structure of this complex in solution (Figure 2A). Interestingly, Goulding *et al*.^8^ have shown that *Mt*FolB is unique among known FolB enzymes for the fact that the apoenzyme is a tetramer in solution. However, when in complex with the reaction product HP (PDB ID 1NBU), *Mt*FolB assumes the basic octameric state of FolB homologs^8^. They presented a substrate-driven oligomerisation model to explain this difference in oligomeric structure, in which conformational changes induced by substrate binding stabilise the tetramer-tetramer interface favouring the formation of the octamer complex. Specifically, they observed that Loop L1 is disordered and loops L2 and L4 have shifted positions in the tetrameric apo form. Additionally, helix α2a is disordered in the tetrameric structure. The octameric structure of *Mt*FolB in complex with 8-MG also presents these major structural changes when compared to the apo-tetrameric form. Loop L1 and the α2a-helix are ordered, while loops L2 and L4 also have shifted positions. Therefore, binding of inhibitor 8-MG to *Mt*FolB induced the major structural changes previously observed upon substrate binding (DHNP), product formation (HP + GA), and complex formation with HP, both in terms of quaternary structure and local rearrangements of loops and helices.

The interaction between *Mt*FolB and 8-MG, located at chain E, was assessed using PoseView^11^ and are summarised in Table S1. The 2D diagram of these interactions are represented in Figure 2(B). 8-MG (**1**) forms hydrogen bonds with 4 *Mt*FolB residues (Asp53E, Tyr54E, Ile73G and Glu74G) (Figure 2B,C). In comparison, when analysing the interactions with the HP located in the chain E using PoseView with PDB ID 1NBU, more interactions were observed. HP forms hydrogen bonds with 6 *Mt*FolB residues (Val18E, Ile73E, Glu74E, Tyr52H, Asp53H, Tyr54H), and makes hydrophobic contacts with Val18E, Leu48H and Tyr54H. Some of these conserved residues, such as Tyr52, Asp53, Tyr54, Ile73 and Glu74, may play an important role in the formation of *Mt*FolB octameric structure. Furthermore, it was previously shown that the residue Tyr54 is essential to aldolase and/or epimerase activities of *Mt*FolB^5^. The octameric structure of *Mt*FolB is suggested as the active form of this enzyme, and 8-MG was previously reported as a non-competitive inhibitor of *Mt*FolB^7^. This interaction may be associated with the inhibition of the enzyme. Although 8-MG is a non-competitive inhibitor capable of binding to both the free enzyme and the enzyme-substrate binary complex, the crystallographic structure obtained shows that 8-MG binds near the active site of *Mt*FolB (Figure 2C). The inhibition traces for 8-MG were hyperbolic while double-reciprocal plots were linear, which suggest that cooperativity plays no role in the enzyme inhibition.

**Figure 3.**
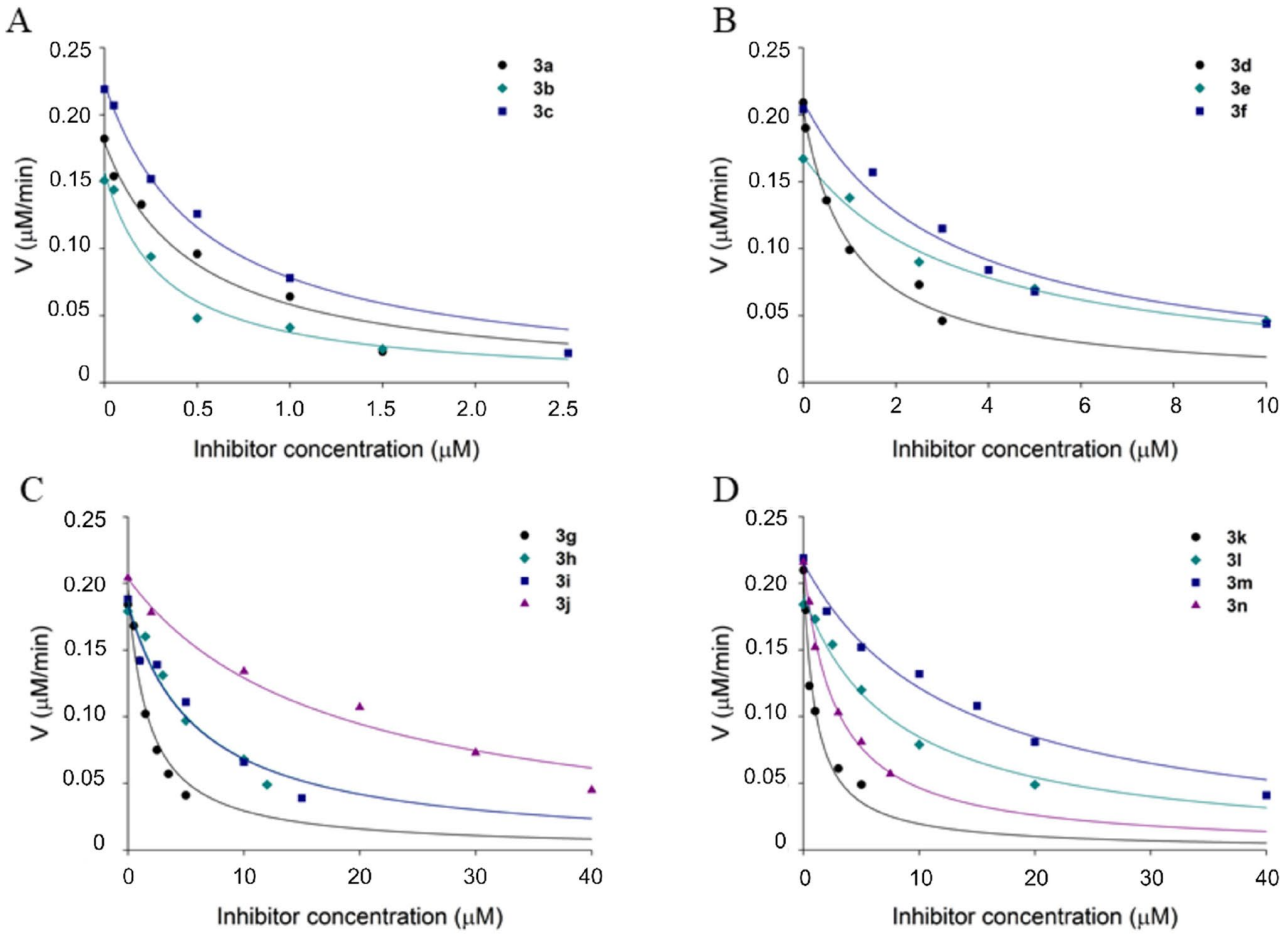
Determination of half-maximum inhibition concentrations (IC_50_ values) of compounds **3a-n** against *Mt*FolB. IC_50_ values were determined performing initial velocity curves of enzymatic activity using 0.3 µM of *Mt*FolB, 1.5 µM of DHNP and various concentrations of inhibitors. The IC_50_ value of each compound provided here were obtained using Equation (1) and plotted using Systat Software SigmaPlot 14.

Fourteen 8-MG (**1**) derivatives were synthesised with 39–84% yields (Scheme 1). These molecules were synthesised in a single reaction step via nucleophilic substitution reactions between 8-MG and bromoacetamides (**2**). The selection of substituents was driven by the goal of evaluating structures incorporating cycloalkyl side chains. This strategic approach aimed to investigate non-planar substituents capable of enhancing complementarity by maximising molecular volume at the binding site on *Mt*FolB. Compounds **3a-n** were evaluated *in vitro* against *Mt*FolB and showed inhibitory activity with IC_50_ values ranging from 0.3 to 17.4 µM (Scheme 1; Figure 3). The inhibition of the enzyme by these compounds was not time-dependent until 40 min of pre-incubation with *Mt*FolB, suggesting that these inhibitors bind to or dissociate from *Mt*FolB quickly (data not shown). Therefore, there is no time dependence for the onset of enzyme inhibition^14^, which warranted the efforts described next.

**Figure 4.**
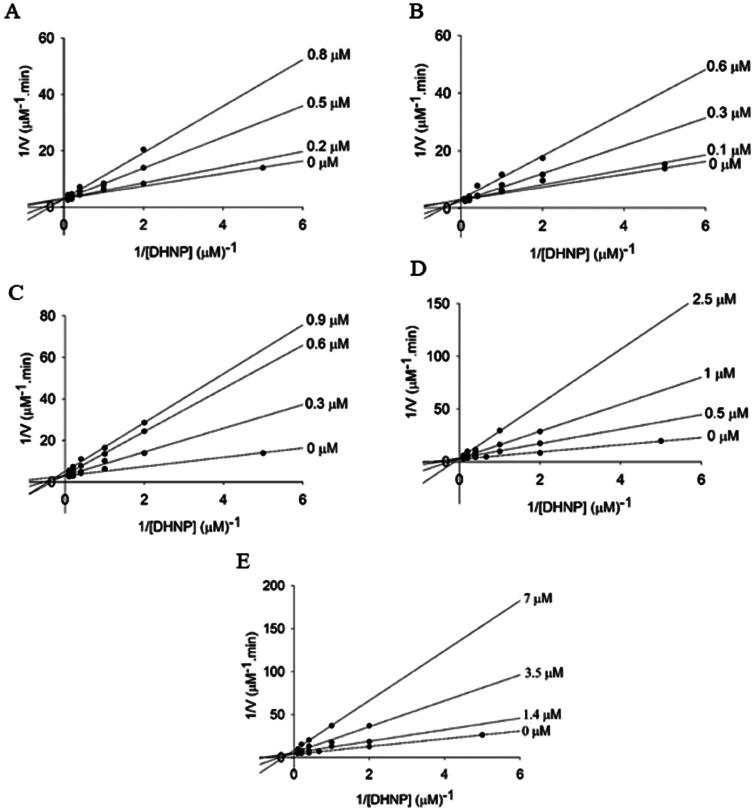
Determination of the inhibition mechanism for compounds **3a-e** on aldolase activity of *Mt*FolB. (A) Compound **3a** (0 – 0.8 µM). The Lineweaver − Burk plot displays a pattern of intersection at the *y*-axis, which indicates competitive inhibition. (B) Compound **3b** (0 – 0.6 µM). The Lineweaver − Burk plot displays a pattern of intersection at the *y*-axis, which indicates competitive inhibition. (C) Compound **3c** (0 – 0.9 µM). The Lineweaver − Burk plot displays a pattern of intersection to the left of the *y*-axis towards DHNP, which is diagnostic of non-competitive inhibition. (D) Compound **3d** (0 – 2.5 µM). The Lineweaver − Burk plot displays a pattern of intersection at the *y*-axis, which indicates competitive inhibition. (E) Compound **3e** (0 – 7 µM). The Lineweaver − Burk plot displays a pattern of intersection to the left of the *y*-axis towards DHNP, which is diagnostic of non-competitive inhibition.

The mode and constant of enzyme inhibition were determined for five selected compounds (**3a**-**e**). Lineweaver − Burk plots were used to suggest the mode of inhibition (competitive, non-competitive or uncompetitive) and the values for the inhibition constants (*K*_is_ and/or *K*_ii_) were determined by fitting the data to appropriate equations^14^. Compounds **3a**, **3b**, and **3d** exhibited competitive inhibition towards the substrate DHNP. This observation is evident in the double reciprocal plot, where the intersecting lines converge at the *y*-axis (Figure 4A,B,D). The *K*_is_ values for these compounds ranged from 0.2 to 0.4 µM (Table 2). Compounds **3c** and **3e** displayed a non-competitive mode of inhibition towards DHNP. The intersecting lines in the double reciprocal plot converge to the left of the *y*-axis and above the *x*-axis (Figure 4C,E), suggesting that these inhibitors bind to both free enzyme and enzyme complexed with substrate, but exhibiting more affinity for the free enzyme. The *K*_is_ and *K*_ii_ values for **3c** were 0.3 and 1.5 µM, respectively; and for **3e** were 1.4 and 6.3 µM, respectively (Table 2). These values, with *K*_is_ < *K*_ii_, support the hypothesis that these compounds have greater affinity for the free enzyme^14^.

**Table 2.** Inhibitory constants and inhibition mode of selected molecules on *Mt*FolB Activity.

Compound	*K*_is_/*K*_ii_ DHNP (µM)	Inhibition mode
3a	0.4 ± 0.1	Competitive
3b	0.3 ± 0.08	Competitive
3c	0.3 ± 0.2 / 1.5 ± 0.9	Non-competitive
3d	0.2 ± 0.05	Competitive
3e	1.4 ± 0.3 / 6.3 ± 1.2	Non-competitive

Molecular docking experiments were performed to suggest the interactions that occur upon enzyme-inhibitor complex formation. Considering that the synthesised compounds were obtained using 8-MG (**1**) as a scaffold, these assays were performed using the crystallographic structure of *Mt*FolB complexed with 8-MG. In this context, possible conformational changes in the enzyme structure due to 8-MG binding could be considered. In these assays, GoldScore was used as the score fitness function, which is taken as the negative of the sum of the component energy terms, so that larger fitness scores are better. The docking scores of the ligands obtained are presented in Table S2. *Mt*FolB complexed with 8-MG inhibitor is a homo-octamer with chains designated as A, B, C, D, E, F, G and H. As reported in the FolB ortholog from *Staphylococcus aureus*^23^, the conserved amino acid residues involved in either substrate binding or catalysis (Glu22, Tyr54, Glu74, and Lys100) are also present in the active site of *Mt*FolB (Glu22, Tyr54, Glu74, and Lys99). Additionally, for *Mt*FolB, the residues Tyr54 and Lys99 play essential roles in the epimerase and aldolase activities of FolB (Figure 1) and it is indispensable for the survival of the bacillus^5^.

**Figure 5.**
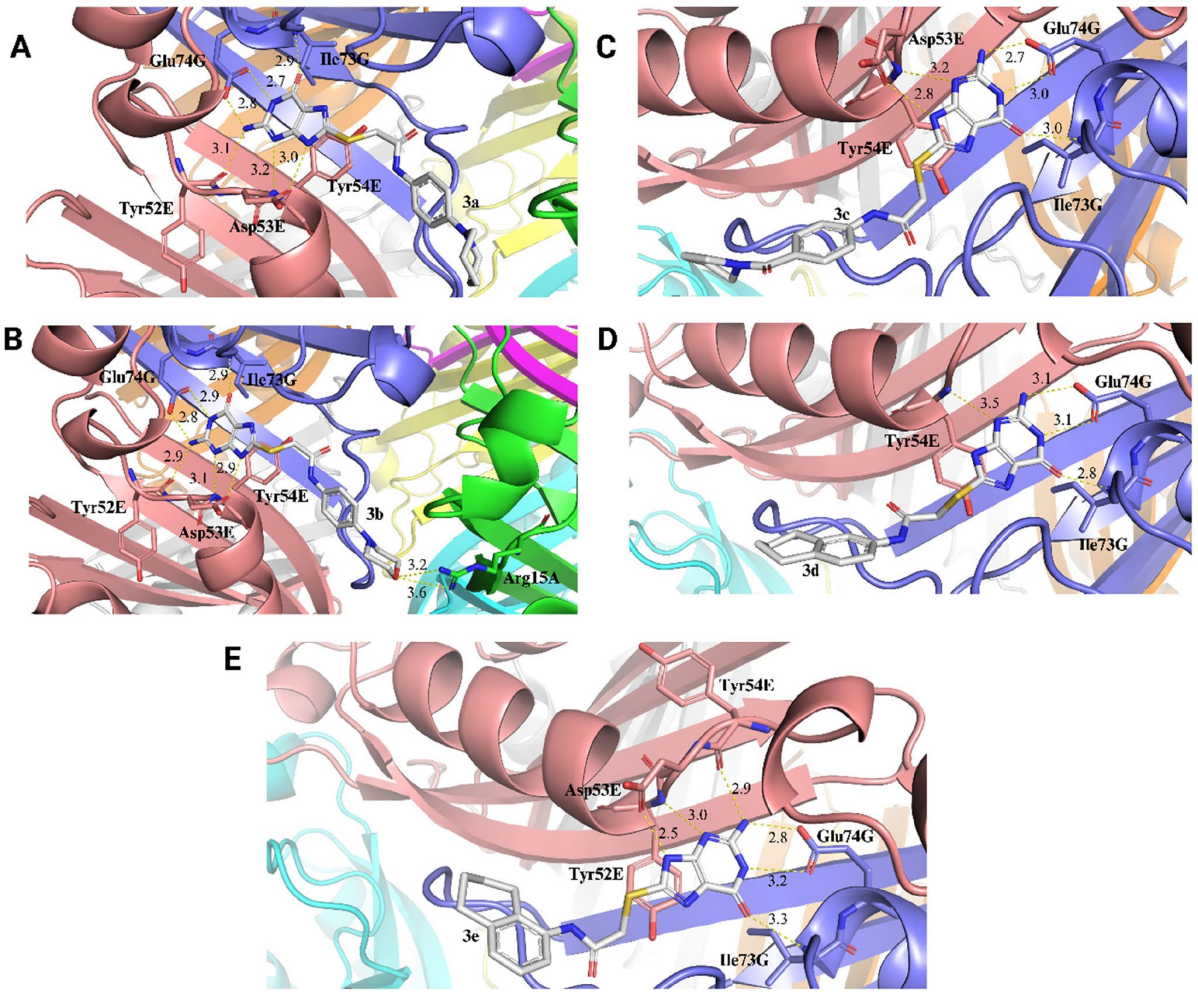
3D interactions between compounds **3a-e** and *Mt*FolB obtained by molecular docking. An enlarged view of 3D interaction between compounds **3a** (A), **3b** (B), **3c** (C), **3d** (D) and **3e** (E) with binding pocket of *Mt*FolB. *Mt*FolB is represented as cartoon, with each chain represented in a distinct colour: A (green), E (salmon), and G (slate blue); the main *Mt*FolB residues that interact by polar contacts with the compounds are visualised as sticks, with carbon atoms coloured according to their respective *Mt*FolB chain, nitrogen atoms in blue, and oxygen atoms in red. Compounds **3a-e** are represented in stick model, with each atom represented in a distinct colour: carbon (gray), oxygen (red), nitrogen (blue), and sulphur (yellow). The distances of hydrogen bonds are provided in Angstrom (Å). Figures were obtained using PyMOL v2.5.

The interactions between selected synthesised compounds (**3a-e**) and *Mt*FolB, obtained through molecular docking simulations, were analysed with PoseView^11^ and PyMOL v2.5^15^ (Figure 5; Table S1; Figure S2). It is remarkable that some residues of the enzyme seem to play a significant role in establishing interactions with these molecules. The residues Tyr54E, Ile73G and Glu74G are involved in interactions with all synthesised compounds, as well the 8-MG (**1**). Interactions were established mainly by hydrogen bonds, π–π stacking, and hydrophobic interactions (Table S1; Figure S2). Compounds **3c**, **3d**, and **3e** establish π–π stacking interactions with Tyr19G. Unlike observed with *Mt*FolB complexed with 8-MG (**1**) (Figure 2B), the synthesised compounds performed hydrophobic contacts with *Mt*FolB, mainly with Ala25A, Tyr54E, Tyr19G, and Pro103G residues (Figure 5; Table S1; Figure S2). Compounds **3b** and **3e** establish hydrophobic contacts with unique additional residues, namely Leu48E and Val18G (**3b**) and Val55E (**3e**). Compound **3b** is also the only 8-MG analogue that perform a hydrogen bond with an additional residue, Arg15A (Figure 5B; Table S1; Figure S2). Interesting, the compound **3e** was found to have a higher *K*_i_ value between the analysed compounds (Table 2), indicating the lowest enzymatic inhibitory potency observed. On the other hand, compound **3d** performed hydrogen bonds just with Tyr54E, Ile73G and Glu74G (Figure 5; Table S1; Figure S2). Importantly, this compound showed a lower *K*_is_ value, corresponding to 0.2 ± 0.05 µM (Table 2), indicating that this molecule is a more potent inhibitor than the scaffold 8-MG (**1**) and the other synthesised derivatives.

All synthesised compounds were assessed for their inhibitory activity on bacillus growth by determining the minimum inhibitory concentration using the H37Rv strain. Compound **3e** exhibited antimycobacterial activity with a MIC value of 40 µg/mL (equivalent to 108 µM), while the other compounds showed no activity against *M. tuberculosis* at the maximum concentration tested. The drug controls in this assay, INH and RIF, exhibited MIC values of 0.31 µg/mL and 0.16 µg/mL (equivalent to 2.3 μM and 0.2 μM), respectively, against the H37Rv strain under the same experimental conditions.

The toxicity of **3e** was thus assessed by evaluating the viability of HepG2 and Vero cell lines following exposure to the compound at a final concentration of 75 µM, 87.5 µM, or 100 µM. The compound was able to maintain the viability of cell lines above 90% at the three concentrations tested (Table 3). Additionally, the aqueous solubility of **3e** was assessed in three conditions: pH 1.2 (simulating stomach), pH 7.4 (simulating plasma) and pH 9.1 (simulating intestine). The molecule exhibited greater solubility at pH 1.2 (612.5 µM) compared to pH 7.4 (1.1 µM) and pH 9.1 (37.6 µM).

**Table 3.** *In vitro* activity of **3e** on viability of HepG2 and Vero cells.

	% of cell viability ± SEM §			
	HepG2		Vero	
Compound	MTT	Neutral Red	MTT	Neutral Red
3e 100 μM	96 ± 2	104 ± 2	94 ± 3	96 ± 2
3e 87.5 μM	97 ± 2	111 ± 3	94 ± 10	102 ± 3
3e 75 μM	108 ± 3	100 ± 6	115 ± 2	96 ± 1

§ Data of three independent experiments were expressed as the means of cell viability ± the standard error of the means. Percent of cell viability measured by MTT and Neutral Red assays, absorbance was measured at 570 nm and 562 nm, respectively. The compound was diluted in DMEM 10–20% FBS with DMSO 0.8% and DMSO 0.8%-treated control wells were considered as 100% of cell viability. One-way ANOVA analysis of variance followed by Dunett’s Multiple Comparison Test post-test using GraphPad Prism 9.0.0 (San Diego, CA, USA).

Despite its high MIC value compared to controls, the compound **3e** demonstrated no apparent toxicity to mammalian cells. The latter warrant further efforts to be pursued using **3e** as a lead compound in the development of new anti-TB drug candidates targeting *Mt*FolB.

## Supplementary Material

Supplemental Material

## Data Availability

The crystal structure of *Mt*FolB complexed with 8-MG was submitted to the Worldwide Protein Data Bank (wwPDB) and is openly available (PDB ID 9B2E).
