# Supplementary material for: Crystal structure of dihydroneopterin aldolase from Mycobacterium tuberculosis associated with 8-mercaptoguanine, and development of novel S8-functionalized analogues as inhibitors: Synthesis, enzyme inhibition, in vitro toxicity and antitubercular activity
Source: J Enzyme Inhib Med Chem. 2024 Aug 14;39(1):2388207. doi: 10.1080/14756366.2024.2388207 (PMC11328599; doi:10.1080/14756366.2024.2388207)
Supplement: Supplemental Material [file IENZ_A_2388207_SM4029.pdf]

Supplementary Tables

Table S1. Interactions between *Mt*FolB and 8-MG (**1**) or selected compounds analyzed with PoseView.

Compound	H bond	π–π stacking interactions	Hydrophobic contact
**1**	Asp53E, Tyr54E, Ile73G, Glu74G	-	-
**3a**	Tyr52E, Asp53E, Tyr54E, Ile73G, Glu74G	-	Ala25A, Tyr54E, Pro103G
**3b**	Arg15A, Tyr52E, Asp53E, Tyr54E, Ile73G, Glu74G	-	Ala25A, Leu48E, Tyr54E, Val18G, Tyr19G
**3c**	Asp53E, Tyr54E, Ile73G, Glu74G	Tyr19G	Ala25A, Tyr54E, Tyr19G, Pro103G
**3d**	Tyr54E, Ile73G, Glu74G	Tyr19G	Ala25A, Tyr54E, Tyr19G
**3e**	Tyr52E, Asp53E, Tyr54E, Ile73G, Glu74G	Tyr19G	Ala25A, Tyr54E, Val55E, Tyr19G

Table S2. Docking scores of the synthesized 8-MG analogues with the *Mt*FolB structure.

Compound	Fitness	Compound	Fitness
**3a**	90.81	**3h**	71.80
**3b**	87.80	**3i**	76.10
**3c**	95.03	**3j**	66.21
**3d**	84.66	**3k**	84.75
**3e**	81.58	**3l**	81.39
**3f**	81.28	**3m**	80.42
**3g**	81.13	**3n**	77.12

Supplementary Figures

Figure S1.

A

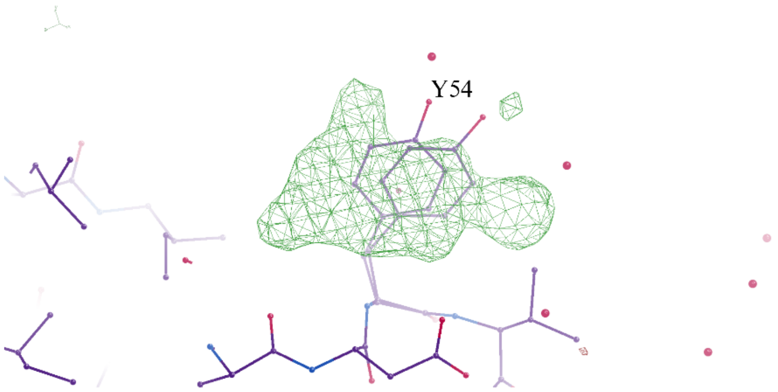

B

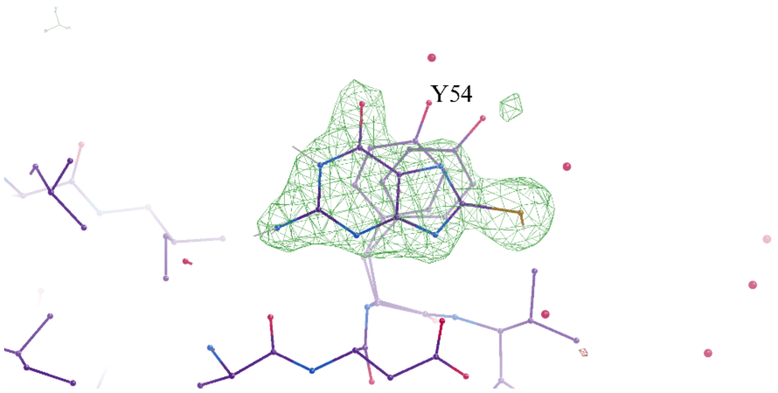

Figure S1. Omit map of the ligand 8-MG. Fo-Fc electron density (green) is contoured to 2 σ, indicating evidence of a bound ligand. The map was created by removing the ligand from the structure and undergoing 3 cycles of gradient energy minimization using Phenix program. The average B-factor for 8-MG is 26.25, while the average B-factors for the residues in the active site is 20.64. The residues considered for the calculation of the B-factor active site average were I5, L48, T51, Y52, D53, Y54, and V55. The image was generated using Coot[1,2].

Figure S2.

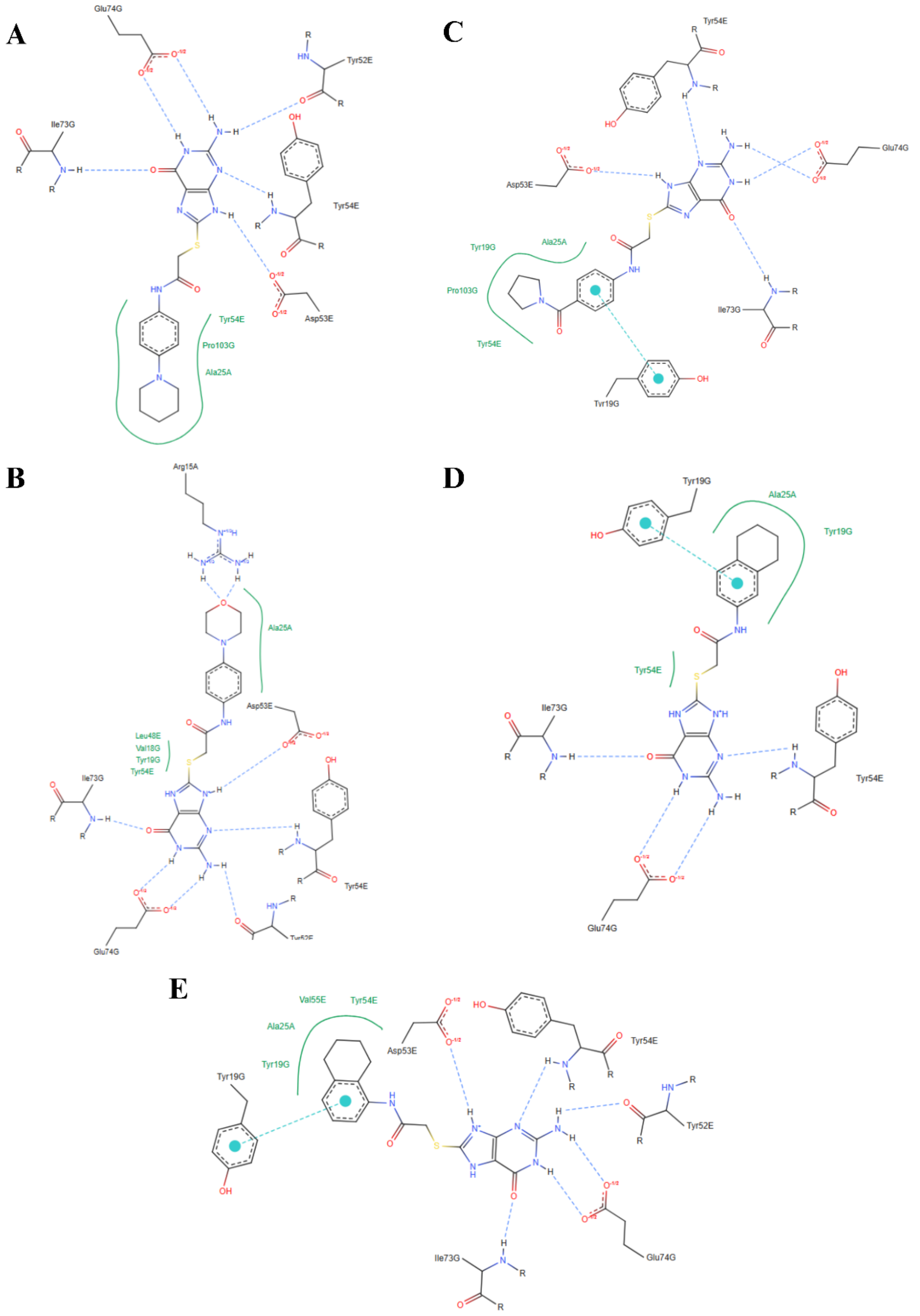

Figure S2. 2D interactions diagrams between compounds **3a-e** and *Mt*FolB residues obtained by molecular docking. Interaction between *Mt*FolB residues and compounds: (A) **3a** (B) **3b** (C) **3c** (D) **3d** (E) **3e**. The hydrogen bonds (blue) and π–π stacking interactions (cyan) are shown in dashed lines, hydrophobic contact are represented by green spline segments which mark hydrophobic regions of the ligand and the corresponding enzyme residue. Figures were obtained using PoseView[3].

Supplementary References

1. Emsley P, Cowtan K. Coot: model-building tools for molecular graphics. Acta Crystallogr D Biol Crystallogr. 2004 Dec;60(Pt 12 Pt 1):2126-32. doi: 10.1107/S0907444904019158. Epub 2004 Nov 26. PMID: 15572765.

2. Emsley P. Tools for ligand validation in Coot. Acta Crystallogr D Struct Biol. 2017 Mar 1;73(Pt 3):203-210. doi: 10.1107/S2059798317003382. Epub 2017 Mar 6. PMID: 28291755; PMCID: PMC5349432.

3. Stierand K, Rarey M. Drawing the PDB: Protein-Ligand Complexes in Two Dimensions. ACS Med Chem Lett. 2010 Aug 31;1(9):540-5. doi: 10.1021/ml100164p. PMID: 24900245; PMCID: PMC4007829.
